# A Unique Case of Lemierre Syndrome Associated with Thrombophilia in an Adult and the Role of Anticoagulation

**DOI:** 10.1155/2010/982494

**Published:** 2010-09-30

**Authors:** Pradeep H. Lakshminarayana, Matthew E. Woodske

**Affiliations:** ^1^Department of Internal Medicine, University of Pittsburgh Medical Center, McKeesport, PA 15132, USA; ^2^Division of Pulmonary, Allergy, and Critical Care Medicine, Montefiore University Hospital, University of Pittsburgh Medical Center, Pittsburgh, PA 15213, USA

## Abstract

Acute septic thrombophlebitis of the internal jugular vein (IJV), better known as Lemierre syndrome, is a rare entity which poses several challenges in management. Treatment involves prompt use of intravenous antibiotics over a prolonged period of time, typically 6–8 weeks. The use of anticoagulation is controversial, but indicated for some. We describe the first reported case of Lemierre syndrome associated with a hypercoagulable state in an adult. We propose that all patients with Lemierre syndrome should be evaluated for hypercoagulable states and that the indications for anticoagulation in Lemierre syndrome are (1) propagation or nonresolution of IJV thrombus despite antibiotics and (2) identification of a hypercoagulable state, as in our case.

## 1. Introduction

Lemierre syndrome is caused by an acute oropharyngeal infection leading to secondary septic thrombosis of the internal jugular vein. The clinical presentation is characteristic, and a cure is possible in most cases with antibiotic therapy. The prevalence of hypercoagulable states in Lemierre syndrome is unknown, with only a few cases reported in pediatric literature. Also, there are no clear indications for use of anticoagulation. We describe the first reported case of Lemierre syndrome associated with a hypercoagulable state in an adult.

## 2. Case Report

**Figure 1:**
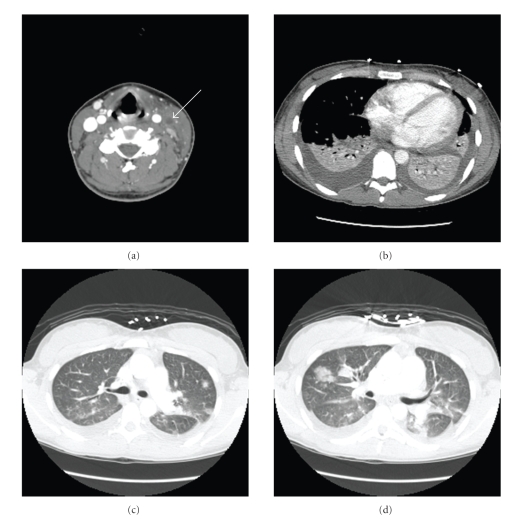
Neck CT scan (a) with contrast reveals left IJV thrombosis noted by the arrow. Chest CT scan reveals bilateral effusions (b) and multiple septic emboli (c and d).

A previously healthy 18-year-old female, presented with sore throat and high grade fever. She further developed pleuritic chest pain, dyspnea and became hypotensive requiring vasopressors. Her examination was significant for left-sided neck tenderness and edema and an enlarged left tonsil without exudates. Initial laboratory investigations revealed leukocytosis with a left shift. CT scan of the neck showed extensive thrombus in the left internal jugular vein (IJV) extending from midthyroid to base of the skull (see Figure 1(a)). The left palatine tonsil had serpentine enhancement with extensive parapharyngeal inflammation. CT thorax showed bilateral pleural effusion (see Figure 1(b)) and multiple septic emboli (see Figures 1(c) and 1(d)). The effusions were exudative and parapneumonic on thoracentesis. Blood cultures were positive for Streptococcus Groups C and G, both of which were penicillin sensitive. After the cultures returned, she was changed from broad spectrum antibiotics to intravenous penicillin G continuous infusion and metronidazole. Because of the persistent left IJV thrombosis despite adequate antibiotics, she was started on intravenous unfractionated heparin. Upon clinical improvement, vasopressors were gradually weaned off, and heparin was transitioned to warfarin to keep the International Normalization Ratio (INR) above 2. 

At the time of diagnosis, she had a thrombophilia screen revealing a heterozygosity for factor V Leiden (FVL) mutation (R506Q, with no haplotype of Factor V HR2 or Cambridge mutations seen) and had a positive Lupus Anticoagulant (LAC) panel (see Table 1). At the time of discharge, metronidazole was stopped, and penicillin was continued intravenously at home via a peripherally inserted central catheter (PICC) for a total duration of 6 weeks. Followup CT scan showed that IJV remained occluded, but septic pulmonary emboli had resolved with no residual cavities. The repeat LAC panel during followup had remained weakly positive (see Table 1). She continues on warfarin and will be evaluated every three months with a LAC panel and repeat CT scan of neck for consideration to stop anticoagulation.

## 3. Discussion

Septic thrombophlebitis of IJV also known as Lemierre syndrome typically presents in healthy teenagers and young adults. The causative organism is usually Fusobacterium necrophorum; however, other causative organisms such as Streptococcus (as in our case) can be found [1]. The pathogenesis involves spread of infection from peritonsillar tissue into lateral parapharyngeal space via lymphatic vessels. Infection of this compartment can cause complications such as thrombophlebitis of IJV and severe sepsis with metastatic infections, including the lungs (septic pulmonary embolism, cavitating pneumonia) and bones (septic osteomyelitis, septic arthritis). The mainstay of treatment is prompt use of intravenous antibiotics over prolonged period of time, typically 6–8 weeks. Commonly used antibiotics include penicillin, clindamycin and metronidazole. 

The prevalence of hypercoagulable states associated with Lemierre syndrome in adults is unknown. There are case reports in children which point to hypercoagulable associations with other forms of suppurative thrombophlebitis. One study showed that five of seven children with acute otitis media associated with venous sinus thrombosis had thrombophilias, including elevated lipoprotein apolipoprotein, antibodies to beta 2-glycoprotein, heterozygous FVL mutation, and homozygous methyltetrahydrofolate reductase (MTHFR) mutation [2]. Other studies have found transient elevations in antiphospholipid antibodies after viral infections in children suggesting an induced hypercoagulable state associated with infectious processes [3]. There is one case report of a child with Lemierre syndrome in association with prothrombin gene mutation, an elevated lipoprotein apolipoprotein and a carrier MTHFR mutation [4]. In our patient, we found an inherited thrombophilia (FVL mutation, heterozygous) and an induced hypercoagulable state, a positive LAC panel which persisted for months after initial presentation. The prevalence of FVL and antiphospholipid antibodies in the general population is reported as less than seven percent for both of these conditions [5]. Given that the incidence of Lemierre syndrome is rare, one case per million population per year by one estimate [6], the association of a hypercoagulable state with Lemierre syndrome is less likely random, and a hypercoagulable state may be a potential risk factor for Lemierre syndrome. 

The role of anticoagulation in Lemierre syndrome is controversial. Case reports and series in other forms of septic thrombophlebitis support the use of anticoagulation. In three case series of patients with pelvic vein thrombophlebitis who failed antibiotic treatment alone, addition of heparin after four to five days of antibiotic use showed defervescence of fever [7]. In another case series, three of six patients with catheter-related central vein septic thrombophlebitis were successfully treated with removal of catheter, antibiotics, and anticoagulation [8]. 

Although no clinical trials exist to examine the role for anticoagulation in Lemierre syndrome, we propose that there are at least two potential indications for anticoagulation in Lemierre syndrome. First, we believe that propagation or nonresolution of the thrombus despite antibiotics is an absolute indication for anticoagulation. One case report showed reduced neurological sequelae seen when anticoagulation was used to treat suppurative cavernous sinus thrombosis in a patient with retrograde propagation from the sigmoid sinus [9]. Second, we believe that anticoagulation should be administered to all patients with any form of predisposing thrombophilia, such as in our patient. Given the rarity of Lemierre syndrome and the impact of correctly identifying a hypercoagulable state, we feel that the possibility of underlying thrombophilia should be explored in every patient with Lemierre syndrome to consider anticoagulation. Because we have no overwhelming evidence to date, anticoagulation in Lemierre syndrome should be considered on an individual basis after weighing the risks and benefits. 

##  Conflict of Interests 

Both authors declare that there is no conflict of interests to disclose.

##  Consent

Patient described in the case report has given consent for the case report to be published.

## Figures and Tables

**Table 1:** Thrombophilia screen.

	Day 4	Day 80	Normal value	Units
PT	17.4	46.4	11–15	seconds
APTT	62.4	47	22–35	seconds
PTT mix	38.5	38.7	28–38	seconds
Thrombin time	76.9	22.9	16–22	seconds
Reptilase time	17.6		14–18	seconds
Protein C activity	17	<10	70–140	percent
Protein S activity	68	<10	58–128	percent
Factor V HR2	ND	ND	—	—
Factor V L M	HZ	HZ	—	—
Factor VIII	2.01	1.05	0.6–1.5	Units/mL
Factor X	0.78	0.1	0.7–1.5	Units/mL
Dil RVVT	1.4	—	0.9–1.3	ratio
A T III activity	46	97	80–120	percent
HLN	Pos	Neg	—	*—*
TTI	1.7	2.2	0.7–1.3	ratio
d APC R	1.8	1.6	2.1–30	ratio
ACL IgG	13.3	11.9	0.0–23	GPL units
ACL IgM	17	8.2	0.0–11	MPL units

## Abbreviations

PT: Prothrombin time
APTT: Activated partial thromboplastin time
PTT mix: Partial thromboplastin time mixed study
Factor V HR2: Haplotype of factor V gene
Factor V L M: Factor V Leiden mutation
HZ: Heterozygous
Dil RVVT: Diluted Russel viper venom time
HLN: Hexagonal lipid neutralization test
TTI: Tissue thromboplastin Index
d APC R: d activated protein C resistance
ACL: Anticardiolipin antibody.
